# Factor Analysis of the BDI-II and HAMD-21 in Patients With Irritable Bowel Syndrome

**DOI:** 10.31083/AP44020

**Published:** 2025-06-19

**Authors:** Akaki Burkadze, Eka Burkadze, Tamar Kandashvili, Teimuraz Silagadze

**Affiliations:** ^1^Department of Psychiatry, Tbilisi State Medical University, 0186 Tbilisi, Georgia; ^2^Department of Scientific Research, Mental Health Clinic “Mental Hub”, 0108 Tbilisi, Georgia; ^3^Department of Internal Medicine, Tbilisi State Medical University, 0186 Tbilisi, Georgia

**Keywords:** depression, irritable bowel syndrome, factor analyses, statistical, principal component analyses, mental health, psychometrics

## Abstract

**Objective:**

The factorial validity of the Beck Depression Inventory-II (BDI-II) and the 21-item Hamilton Depression Rating Scale (HAMD-21) in individuals with Irritable Bowel Syndrome (IBS) has not yet been investigated. This study aimed to assess the factor structure of these instruments and analyze their interrelationships within the Georgian IBS population.

**Methods:**

Principal component analysis was performed on data from 89 IBS patients. Factors were determined using eigenvalues ≥1, with factor loadings exceeding 0.4 and oblique rotations identifying factor compositions. The Kaiser-Meyer-Olkin measure, Cronbach’s alpha, Bartlett’s test, communality, non-redundant residuals, and the component correlation matrix were used to assess factor validity. Intercorrelations between the scales’ symptoms were also analyzed.

**Results:**

The mean BDI-II score was 24.31 (standard deviation (SD) = 14.93) and the mean HAMD-21 score was 17.38 (SD = 8.91). According to the cutoff criteria for both scales, the sample exhibited moderate depression. The BDI-II identified three factors, while the HAMD-21 revealed four distinct factors. Combined analysis showed that most BDI-II items and core depressive symptoms from HAMD-21 clustered into Component I. Component II included four HAMD-21 items: insomnia (delayed), agitation, somatic anxiety, and insight. Significant positive correlations between paired BDI-II and HAMD-21 symptoms were found, with a high correlation (r = 0.88, *p* = 0.000) between the scales, differing from previous findings.

**Conclusion:**

The high correlations within components, along with low non-redundant residuals and high communality, indicate satisfactory factor validity for both the BDI-II and HAMD-21. The self-report BDI-II inventory and the HAMD-21 scale are complementary in evaluating depressive symptoms in patients with IBS.

## Main Points

1. The factorial validity of the HAMD-21 and BDI-II in patients with IBS has not yet been explored.

2. We examined the factor structure of the HAMD-21 and BDI-II in IBS patients.

3. The factorial validity of the HAMD-21 and BDI-II in IBS patients is satisfactory.

4. The factors identified in this study are consistent with those reported in prior research.

5. Self-reports and clinician ratings are complementary rather than redundant.

## 1. Introduction

Irritable Bowel Syndrome (IBS) is among the most prevalent functional gastrointestinal (GI) disorders, characterized by abdominal pain and discomfort, along with altered bowel habits, which may include predominantly diarrhea (IBS-D), constipation (IBS-C), or a combination of both (IBS-M) [1, 2, 3]. IBS impacts 5%–10% of people worldwide, with significant variations in prevalence across different countries and within various cultures [4, 5]. Recent systematic reviews and meta-analyses suggest that these variations are not due to inconsistent diagnostic criteria or methodological differences but rather reflect genuine global differences [5].

Evidence shows that IBS is associated with a poor quality of life and diminished social functioning [1]. Significant psychosocial issues, including depression, have been reported in 50%–60% of IBS patients [6, 7, 8]. The coronavirus disease 2019 (COVID-19) pandemic significantly contributed to a rise in depression, with global prevalence increasing by about 28% in 2020 [5]. IBS is also among the various GI manifestations of post-acute COVID-19 syndrome [9].

In light of the potential rise in IBS prevalence alongside the growing global burden of mental health disorders, it is crucial to address how current therapies can be adapted to manage patients with co-occurring conditions effectively. Proper identification and treatment of depression can enhance quality of life, emphasizing the need for valid depression assessment tools.

The Beck Depression Inventory (BDI), developed by Aaron T. Beck and his colleagues in 1961, quickly became a cornerstone for evaluating depression. The BDI-II used in our study is a revised version from 1966 that aligns with the Diagnostic and Statistical Manual of Mental Disorders Fourth Edition (DSM-IV) criteria [10, 11, 12]. It includes 21 items assessing various depression symptoms, with scores ranging from 0 to 63. A total score of 0–13 typically indicates minimal depression, 14–19 suggests mild depression, 20–28 reflects moderate depression, and 29–63 signifies severe depression [10].

The Hamilton Depression Rating Scale (HAMD) is the most widely used clinician-administered assessment for depression. Developed in 1960, it provides a standardized method for diagnosing depression and monitoring changes over time. Despite Max Hamilton’s acknowledgment that there was “room for improvement” in the HAMD, the scale’s core structure has remained largely unchanged for 60 years [13]. The HAMD-21 includes 21 items assessing symptoms and functional impairments, with scores ranging from 0 to 7 indicating no depression, 8–13 suggesting mild depression, 14–18 reflecting moderate depression, and 19 or higher signifying severe depression [13].

Over the years, numerous studies have demonstrated the reliability and validity of both the BDI and the HAMD [12, 13, 14, 15]. However, none of these studies have specifically examined the factor structure of these scales within the IBS population. Factor analyses of the HAMD and BDI conducted in the general population may not be readily applicable to the IBS population due to symptom overlap between IBS and depression, as well as confounding variables such as medication use, dietary habits, comorbid conditions, and other IBS-specific factors [1].

Moreover, the majority of the evidence on IBS and co-morbid depression comes from high-income countries, particularly in Western Europe and North America, with very little clinical or academic research on the manifestations of depression in IBS patients in the South Caucasus region. Given that socio-cultural differences can affect the manifestation of mental disorders, findings from research conducted in Western countries may not be applicable.

The study aimed to examine the factor structure of two widely recognized instruments, the BDI-II and HAMD-21, and to analyze the relationships between them within the Georgian IBS population.

## 2. Materials and Methods

A prospective observational study was carried out in the Gastroenterology department of the V. Iverieli Endocrinology Metabolism Dietology Center “Enmedic”, a secondary specialist unit located in the capital city of Tbilisi (population 1,175,200). This center serves the country (population 3,716,900) by providing outpatient services. All patients aged 18 years and older with IBS who were admitted to the outpatient setting from January 1, 2020 through December 31, 2023 were eligible for participation.

A total of 287 patients suspected of having IBS were examined and 89 of these patients were selected for study participation based on a confirmed diagnosis. The diagnosis of IBS was based on the Rome IV criteria and the Bristol Stool Form Scale was used to determine the clinical form of IBS. Subjects with organic GI diseases, clinical cases with any red flag criteria, a positive Helicobacter pylori stool antigen test, or clinically significant chronic diseases (such as cardiovascular, respiratory, liver, kidney issues, or uncontrolled diabetes) were excluded. Patients were also excluded if they reported conditions that would limit the use of esophagogastroduodenoscopies, such as malignant tumors of any location, pregnancy, or lactation. Additionally, patients who had undergone any surgical operation in the past 6 months, had post-colectomy, inflammatory or obstructive bowel disease, or celiac disease were excluded. Seven patients (2.4%) opted out of participating in the study.

All patients underwent a comprehensive GI and psychological assessment. Of the 89 patients with IBS, 57 (64.04%) underwent gastroscopic examination and 32 (35.96%) underwent GI X-ray studies with barium sulfate specifically to examine the esophagus and stomach. Additionally, 44 (49.44%) patients underwent colonoscopy, which allowed us to rule out organic GI disorders. All patients were tested for *Helicobacter pylori* antigen in stool; all patients included in the study tested negative, which was a necessary criterion for inclusion.

Communication with the patients was conducted by the medical team. Patients were informed about the study and gave written consent to participate. The study protocol received approval from the Ethics Review Committee (N1267251.25/11/2021).

GI symptoms were assessed using the IBS symptom questionnaire, which included questions about diet, primary intestinal symptoms, defecation-related symptoms, medication intake, and extra-intestinal symptoms, as shown in **Supplementary Material 1**. This questionnaire was completed during a face-to-face interview.

Depression was assessed using the HAMD-21 and BDI-II. The HAMD-21, administered by trained professionals through conversation and observation, covers a range of depressive symptoms, including affective, cognitive, and somatic aspects. Item scores range from 0 to 4 (absent to severe) or from 0 to 2 (absent to clearly present).

Participants completed the self-administered BDI-II, selecting statements that best described their feelings over the past 2 weeks, including the current day. Responses were scored on a 0–3 scale, with higher scores indicating greater symptom severity. The BDI-II and HAMD-21 were both used to assess the patient’s condition for the “past week, including today”.

### Statistical Analyses

Pearson product-moment correlations were calculated among the 21 BDI-II and 21 HAMD symptoms. To determine whether the resultant correlation matrix was suitable for principal components analysis, the Kaiser-Meyer-Olkin (KMO) Measure of Sampling Adequacy was calculated. A KMO value greater than 0.8 was considered adequate. The internal consistency of the scales was assessed using Cronbach’s alpha (α). Bartlett’s Test of Sphericity was conducted to confirm that there was sufficient redundancy between the variables that could be summarized with a smaller number of factors. We used an eigenvalue ≥1, in combination with Cattell’s scree plots, to determine the number of appropriate components to retain for subsequent analyses. Because previous research indicated a high degree of correlation between the factors, an oblique (direct oblimin) rather than orthogonal rotation was applied. Factor loadings with an absolute value greater than 0.4, accounting for approximately 16% of the variance in the variable, were considered. When items loaded above 0.4 on multiple factors, the factor chosen was based on the clinically most plausible interpretation. We assessed the percentage of non-redundant residuals and compared the pattern and structure matrices to examine the impact of shared variance. Communalities were computed to indicate the proportion of variance explained by the extracted factors. The correlations between the scales were evaluated using Pearson’s correlation coefficient. All analyses were performed using version 28.0 of the Statistical Package for the Social Sciences (SPSS, IBM Corp., Chicago, IL, USA) software.

Although the BDI-II is reported to have three generalizable factors and the HAMD-21 four factors, we chose to perform separate principal components analyses to assess whether our data would corroborate the symptom clusters described for each instrument [2].

## 3. Results

In the total sample of 89 patients, females comprised 59.6% (n = 53), resulting in a male-to-female ratio of 1:1.5. The age of patients ranged from 25 to 78 years, with a median age of 52 years and a mode of 38 years. The mean age was 51.4 (SD = 14.67) years. The modal age group was 25–44 years, accounting for 38 patients (42.7%), followed by the 45–64 years group, which accounted for 33 patients (37.1%).

All patients (100%) reported taking proton pump inhibitors. Fifty-two patients (58.43%) used selective histamine type 2 receptor antagonists (H2 blockers) and 82 patients (92.13%) used prokinetics. Forty-two patients (47.19%) reported taking laxatives and 21 patients (23.6%) confirmed the use of antidiarrheal medications.

Due to bothersome symptoms, 82 patients (92.13%) used analgesics/painkillers. Antidepressants were reported by 25.84% of patients and probiotics were used by 71.91% of study participants. At the time of recruitment, participants reported not having taken any medication during the previous 4 weeks. They noted the unsystematic nature of previous treatments, which were characterized by partial, low, or transitional efficacy. In some cases, patients had stopped taking medication, and for those who continued, symptoms persisted.

The GI complaint questionnaire revealed that 62 patients (69.66%) did not follow a nutritional regimen/diet and primarily ate irregularly, one to two, or three times a day. Fifty-three patients (59.55%) did not consume fiber-rich, plant-based food. Abdominal discomfort was confirmed by all patients (100%), meteorism was reported by 86 patients (96.63%), and spasmodic pain in the abdominal area at least two to three times a week was reported by 76 patients (85.39%). Pain or discomfort in the abdominal area, which improved after defecation, was experienced by 48.31% (n = 43) of patients. Twenty-four patients (26.97%) were classified as having constipation-predominant IBS (IBS-C), 19 patients (21.35%) as having diarrhea-predominant IBS (IBS-D), and 46 patients (51.69%) as having mixed IBS (IBS-M). Worsening of symptoms after meals was reported by 72 patients (80.90%). Almost all symptoms were present simultaneously in 82 patients (92.13%).

The mean BDI-II score was 24.31 (SD = 14.93), while the mean HAMD score was 17.38 (SD = 8.91). Table 1 shows that 37.1% (n = 33) of IBS patients experienced severe depression, 7.9% (n = 7) had moderate depression, and 27% (n = 24) had mild depression, resulting in an overall prevalence of 71.9% according to the BDI-II. In comparison, the HAMD results revealed that 22.5% (n = 20) of patients had mild depression, 13.5% (n = 12) had moderate depression, and 34.8% (n = 30) experienced severe depression, yielding an overall prevalence of 70.8%.

**Table 1.** **Depression Levels According to the Beck Depression Inventory-II and Hamilton Depression Rating Scale in Patients with Irritable Bowel Syndrome**.

Depression Level	BDI-II (n = 89)	HAMD-21 (n = 89)
Severe Depression	37.1% (n = 33)	34.8% (n = 30)
Moderate Depression	7.9% (n = 7)	13.5% (n = 12)
Mild Depression	27% (n = 24)	22.5% (n = 20)
Overall Prevalence	71.90%	70.80%

BDI-II, Beck Deprasion Inventory-II; HAMD-21, 21 item Hamilton Depression Rating Scale.

### 3.1 BDI-II Structure

Cronbach’s alpha (α) was 0.96, demonstrating strong reliability for the self-rated BDI-II. A KMO value of 0.92 confirms excellent sampling adequacy. Bartlett’s test of sphericity revealed that correlations between items were sufficiently large (χ^2^(210) = 1633.510, *p* = 0.000). A preliminary analysis was performed to calculate the eigenvalues for each component in the data. The scree test identified three distinct components, each with eigenvalues greater than Kaiser’s criterion of 1, collectively accounting for 71.09% of the variance. Table 2 presents the factor loadings after rotation for the three-factor solution derived from Kaiser’s criterion. Only item 14 (Worthlessness) exhibited more than one factor loading greater than 0.4. In line with the clinically most plausible solution, this item was assigned to Component I.

**Table 2.** **Principal component loadings for the Beck Depression Inventory-II**.

Symptom	Rotated component loadings			Communalities
	I	II	III	
1. Sadness	**0.57**	0.18	–0.29	0.77
2. Pessimism	**0.94**	0.07	0.18	0.72
3. Past Failure	**0.93**	0.01	0.05	0.82
4. Loss of Pleasure	**0.83**	0.06	–0.06	0.79
5. Guilty Feelings	**0.78**	–0.03	–0.11	0.72
6. Punishment Feelings	**0.80**	0.01	0.03	0.62
7. Self-Dislike	**0.60**	0.08	–0.29	0.72
8. Self-Criticalness	**0.40**	0.28	–0.36	0.70
9. Suicidal Thoughts or Wishes	0.25	0.08	**–0.61**	0.69
10. Crying	0.28	–0.08	**–0.64**	0.68
11. Agitation	0.11	0.21	**–0.57**	0.55
12. Loss of Interest	**0.76**	–0.06	–0.15	0.73
13. Indecisiveness	0.28	0.28	**–0.55**	0.81
14. Worthlessness	**0.44**	–0.24	**–0.51**	0.66
15. Loss of Energy	**0.53**	0.03	–0.31	0.63
16. Changes in Sleeping Pattern	0.20	0.12	**–0.67**	0.75
17. Irritability	0.15	**0.77**	0.02	0.69
18. Changes in Appetite	–0.10	**0.88**	–0.04	0.74
19. Concentration Difficulty	0.37	0.17	**–0.51**	0.77
20. Tiredness or Fatigue	–0.11	–0.12	**–0.91**	0.66
21. Loss of Interest in Sex	–0.09	0.21	**–0.82**	0.72
Eigenvalues	12.44	1.37	1.11	
% of Variance	59.25%	6.55%	5.29%	

n = 89: Component loadings greater than 0.4 are highlighted in bold. The results of the pattern analysis are presented. Extraction Method: Principal Component analyses. Rotation Method: Oblimin with Kaiser Normalization.

The scree test identified three distinct components, interpreted as representing (I) cognitive distortions, (II) behavioral, and (III) somatic complaints. Component I had its three highest loadings with items 2 (Pessimism), 3 (Past Failure), and 4 (Loss of Pleasure), whereas Component II was represented solely by items 17 (Irritability) and 18 (Changes in Appetite). Component III’s three highest loadings were items 20 (Tiredness or Fatigue), 21 (Loss of Interest in Sex), and 16 (Changes in Sleeping Pattern). Only one symptom, item 8 (Self-Criticalness), failed to load significantly on any component.

The correlation matrix revealed that several items exhibited strong correlations with one another. Only one item, 18 (Changes in Appetite), was found to be weakly correlated with other items (r < 0.3, *p* =0.000). To evaluate the model’s fit, we examined the differences between the observed correlations and those predicted by the model, utilizing the reproduced matrix. An effective model should have fewer than 50% of non-redundant residuals with absolute values exceeding 0.05. Our model demonstrated that 33% of non-redundant residuals had absolute values greater than 0.05.

Communality (h^2^) reflects the proportion of shared variance within a variable. A value above 0.6 is considered ideal. In our sample, the average communality was 0.71. Only one item, 11 (Agitation), had a value below 0.6 (h^2^ = 0.554).

### 3.2 HAMD-21 Structure

Cronbach’s alpha (α) was 0.90, indicating strong internal consistency of the total HAMD scale. A KMO value of 0.85 demonstrates excellent sampling adequacy. Bartlett’s test of sphericity indicated that correlations between items were sufficiently large (χ^2^(210) = 911.202, *p* = 0.000). The scree test identified six distinct components with eigenvalues greater than Kaiser’s criterion of 1, collectively accounting for 68.56% of the variance. Table 3 shows the factor loadings after rotation for the six-factor solution according to Kaiser’s criterion. Items 11 (Anxiety-Somatic), 12 (Somatic Symptoms-Gastrointestinal), and 14 (Genital Symptoms) had more than one-factor loading >0.4 (*p* = 0.000). Based on the clinically most plausible solution, the decision was made to place: 11 (Anxiety-Somatic) in Component II, and 12 (Somatic Symptoms-Gastrointestinal) and 14 (Genital Symptoms) in Component III.

**Table 3.** **Principal Component Loadings for the Hamilton Depression Rating Scale**.

Symptom	Rotated component loadings						Communalities
	I	II	III	IV	V	VI	
1. Depressed Mood	**0.75**	–0.21	–0.03	0.02	–0.04	0.06	0.69
2. Feelings of Guilt	**0.85**	–0.14	0.08	0.00	0.14	0.10	0.76
3. Suicide	**0.78**	–0.08	–0.08	0.11	0.08	0.07	0.71
4. Insomnia-Initial	**0.53**	–0.05	–0.19	0.06	–0.35	0.24	0.74
5. Insomnia-Middle	**0.78**	–0.12	0.11	–0.06	–0.18	0.10	0.73
6. Insomnia-Delayed	–0.01	0.02	–0.08	–0.05	**–0.88**	–0.12	0.78
7. Work and Interest	**0.79**	–0.02	0.09	0.06	0.08	0.02	0.59
8. Retardation	**0.68**	0.11	0.04	0.00	–0.14	–0.13	0.47
9. Agitation	–0.23	**0.68**	–0.15	0.20	0.13	–0.06	0.59
10. Anxiety-Psychic	**0.62**	–0.13	–0.18	0.10	–0.02	0.29	0.74
11. Anxiety-Somatic	**0.61**	**0.41**	–0.14	–0.09	–0.19	–0.15	0.65
12. Somatic Symptoms-Gastrointestinal	**0.46**	0.02	–0.41	–0.33	0.36	–0.18	0.66
13. Somatic Symptoms-General	–0.22	0.10	**–0.84**	–0.05	–0.03	–0.07	0.67
14. Genital Symptoms	**0.42**	0.24	–0.40	0.24	0.04	0.27	0.68
15. Hypochondriasis	**0.84**	0.16	0.01	0.21	–0.06	–0.07	0.78
16. Weight Loss	**0.59**	–0.01	–0.18	–0.36	0.05	0.24	0.67
17. Insight	0.16	**0.62**	0.12	–0.11	–0.34	0.29	0.66
18. Diurnal Variation	0.10	–0.35	**–0.51**	0.10	–0.30	0.03	0.56
19. Depersonalization and Derealization	–0.09	0.12	0.11	–0.08	0.09	**0.92**	0.81
20. Paranoid Symptoms	0.27	–0.27	–0.25	0.22	–0.05	**0.51**	0.72
21. Obsessional Symptoms	0.18	0.06	0.01	**0.86**	0.05	–0.05	0.76
Eigenvalues	7.78	1.63	1.50	1.19	1.12	1.05	
% of Variance	37.53%	7.76%	7.15%	5.71%	5.36%	5.03%	

n = 89. Component loadings greater than 0.4 are highlighted in bold. The results of the pattern analysis are presented. Extraction Method: Principal Component analyses. Rotation Method: Oblimin with Kaiser Normalization.

The results indicated that Component I had its three highest loadings on items 2 (Feelings of Guilt), 15 (Hypochondriasis), and 7 (Work and Interest). Component II had only three salient loadings: 9 (Agitation), 17 (Insight), and 11 (Anxiety-Somatic). Component III clustered items 13 (Somatic Symptoms-General), 18 (Diurnal Variation), and 12 (Somatic Symptoms-Gastrointestinal) together. Component IV had a single salient loading for item 21 (Obsessional Symptoms), while item 6 (Insomnia-Delayed) loaded on Component V. Items 19 (Depersonalization and Derealization) and 20 (Paranoid Symptoms) loaded saliently on Component VI.

As a factor requires a minimum of two items, and since Components IV and V each consist of only one item, the total number of genuine factors is four.

The correlation matrix indicates that several items have strong correlations (r > 0.4, *p* = 0.000). Variables 6 (Insomnia-Delayed), 9 (Agitation), 11 (Anxiety-Somatic), 12 (Somatic Symptoms-Gastrointestinal), and 19 (Depersonalization and Derealization) all have correlation coefficients below 0.4 (*p* = 0.000). The reproduced matrix shows 38% of non-redundant residuals with absolute values greater than 0.05. In our sample, the average communality was 0.68.

### 3.3 Combined BDI-II and HAMD-21 Structure

To test the hypothesis that both instruments capture a single underlying construct of depression, we performed a combined analysis of all 42 items.

A KMO value of 0.94 indicates excellent sampling adequacy. Bartlett’s Test of Sphericity suggested that the correlations among items were sufficiently large (χ^2^(861) = 8494, *p* = 0.000). The scree test revealed four significant dimensions underlying the intercorrelations among the 21 BDI-II and 21 HAMD items. A brief review of the percentage of variance explained by the correlation matrix indicates that Component I accounted for 58.77% of the common variance between the items of the two instruments, Component II accounted for 5.35%, and Component III explained 3.47%. Thus, Component I emerged as the most significant dimension underlying the relationship between the BDI-II and HAMD-21 items. In total, the four components accounted for 70.73% of the overall variance between the BDI-II and HAMD-21.

Table 4 displays the oblique-rotated principal components for the symptoms of the BDI-II and HAMD-21, highlighting salient component loadings greater than 0.4 (*p* = 0.000) in bold. Component I had 20 salient loadings from the BDI-II symptoms and 13 from the HAMD-21 symptoms. Component II featured four salient HAMD symptoms, while no BDI-II symptoms were represented. The HAMD symptoms loading on Component II were 6 (Insomnia-Delayed), 11 (Anxiety-Somatic), 9 (Agitation), and 17 (Insight). Component III had one salient loading (Changes in Appetite) from the BDI-II and three salient loadings (Somatic Symptoms-General, Diurnal Variation, and Obsessional Symptoms) from the HAMD. With regard to component IV, there were no salient loadings from the BDI-II, while the HAMD-21 included one significant loading: 12 (Somatic Symptoms-Gastrointestinal).

**Table 4.** **Principal Component Loadings for the Beck Depression Inventory and the Hamilton Depression Rating Scale**.

Symptom	Rotated component loadings				Communalities
	I	II	III	IV	
Beck Depression Inventory-II					
1. Sadness	**0.83**	–0.05	–0.15	0.05	0.79
2. Pessimism	**0.72**	–0.24	–0.20	–0.02	0.66
3. Past Failure	**0.87**	–0.20	–0.11	0.05	0.81
4. Loss of Pleasure	**0.78**	–0.17	–0.25	0.01	0.81
5. Guilty Feelings	**0.78**	–0.12	–0.23	–0.02	0.79
6. Punishment Feelings	**0.74**	–0.13	–0.21	0.03	0.71
7. Self-Dislike	**0.86**	–0.14	–0.11	0.03	0.81
8. Self-Criticalness	**0.82**	0.01	–0.11	0.09	0.76
9. Suicidal Thoughts or Wishes	**0.91**	0.02	0.13	0.01	0.74
10. Crying	**0.80**	–0.05	–0.09	–0.05	0.71
11. Agitation	**0.61**	0.12	–0.31	0.05	0.69
12. Loss of Interest	**0.88**	–0.05	–0.02	–0.02	0.79
13. Indecisiveness	**0.81**	0.06	–0.17	0.04	0.83
14. Worthlessness	**0.87**	–0.03	0.11	–0.09	0.71
15. Loss of Energy	**0.84**	0.05	0.01	0.07	0.71
16. Changes in Sleeping Pattern	**0.83**	0.21	0.14	0.06	0.71
17. Irritability	**0.41**	0.20	–0.52	0.15	0.74
18. Changes in Appetite	0.31	0.06	**–0.69**	0.16	0.79
19. Concentration Difficulty	**0.93**	0.01	0.05	0.06	0.81
20. Tiredness or Fatigue	**0.59**	0.11	–0.34	–0.14	0.77
21. Loss of Interest in Sex	**0.63**	0.13	–0.31	–0.01	0.74
Hamilton Depression Rating Scale					
1. Depressed Mood	**0.84**	0.03	0.04	–0.02	0.69
2. Feelings of Guilt	**0.82**	–0.07	–0.17	–0.03	0.82
3. Suicide	**0.91**	0.02	0.13	0.04	0.74
4. Insomnia-Initial	**0.74**	0.33	0.14	–0.10	0.70
5. Insomnia-Middle	**0.85**	0.06	0.28	–0.17	0.67
6. Insomnia-Delayed	0.16	**0.70**	0.02	–0.15	0.60
7. Work and Interest	**0.77**	0.08	–0.02	–0.05	0.66
8. Retardation	**0.65**	0.26	0.03	0.05	0.53
9. Agitation	–0.37	**0.58**	–0.25	0.11	0.43
10. Anxiety-Psychic	**0.76**	0.09	–0.11	–0.08	0.74
11. Anxiety-Somatic	**0.45**	**0.59**	0.05	0.07	0.63
12. Somatic Symptoms-Gastrointestinal	0.29	0.01	–0.07	**0.87**	0.79
13. Somatic Symptoms-General	0.17	0.18	**–0.71**	–0.11	0.77
14.Genital Symptoms	**0.50**	0.24	–0.35	–0.07	0.70
15. Hypochondriasis	**0.75**	0.21	–0.11	–0.06	0.79
16. Weight Loss	**0.76**	0.03	–0.07	0.20	0.64
17. Insight	0.26	**0.44**	–0.13	–0.26	0.49
18. Diurnal Variation	0.34	0.14	**–0.57**	–0.14	0.74
19. Depersonalization and Derealization	0.38	–0.05	–0.31	–0.38	0.54
20. Paranoid Symptoms	**0.63**	–0.04	–0.27	–0.22	0.71
21. Obsessional Symptoms	0.18	0.12	**–0.46**	–0.35	0.54
Eigenvalues	24.68	2.25	1.45	1.31	
% of Variance	58.77%	5.35%	3.47%	3.12%	

n = 89. Component loadings greater than 0.4 are highlighted in bold. The results of the pattern analysis are presented. Extraction Method: Principal Component analyses. Rotation Method: Oblimin with Kaiser Normalization.

The Pearson product-moment correlation between the BDI-II and HAMD-21 was 0.88 (*p* = 0.000).

### 3.4 Similar Symptom Correlations

The correlations between individual items with similar content from both instruments were assessed. Seven symptom pairs were identified as having comparable content: (1) Sadness/Depressed Mood, (2) Guilt/Feeling of Guilt, (3) Suicidal Ideas/Suicide, (4) Changes in Sleeping Pattern/Insomnia Delayed, (5) Loss of Interest in Sex/Genital Symptoms, (6) Changes in Appetite/Somatic Symptoms-Gastrointestinal, and (7) Agitation/Agitation.

As shown in Table 5, with correlation coefficients ranging from 0.25 to 0.88, four symptom pairs (Sadness/Depressed Mood, Guilt/Feeling of Guilt, Suicidal Ideas/Suicide, Loss of Interest in Sex/Genital Symptoms) revealed highly positive correlations (*p* = 0.000). One symptom pair (Agitation/Agitation) revealed a moderately positive correlation (r = 0.63, *p* = 0.000), one pair (Changes in Sleeping Pattern/Insomnia Delayed) showed a lower positive correlation (r = 0.43, *p* = 0.000), and one pair (Changes in Appetite/Somatic Symptoms-Gastrointestinal) exhibited a negligible correlation (r = 0.26, *p* = 0.000). The name of the BDI-II symptom is shown first in each pair, followed by the corresponding HAMD-21 name.

**Table 5.** **Correlations Between BDI-II and HAMD-21 Items Measuring Similar Symptoms**.

Symptom	BDI-II Item No.	HAMD-21 item No.	r
Sadness/Depressed Mood	1	1	0.83^*^
Guilt/Feeling of Guilt	5	2	0.87^*^
Suicidal Ideas/Suicide	9	3	0.89^*^
Changes in Sleeping Pattern/ Insomnia Delayed	16	6	0.43^*^
Loss of Interest in Sex/Genital Symptoms	21	14	0.88^*^
Changes in Appetite/Somatic Symptoms Gastrointestinal	18	12	0.26^*^
Agitation/Agitation	11	9	0.63^*^

n = 89. ^*^p < 0.001.

## 4. Discussion

It is crucial to acknowledge that multiple IBS-related symptoms, such as fatigue, sleep disturbances, changes in appetite, physical discomfort, and concentration difficulties, significantly overlap with symptoms of depression. Therefore, there is a need for more precise and individualized approaches to managing both IBS and depression, including the selection of appropriate data collection tools.

There is an ongoing discussion about whether self-report and clinician-rated depression scales assess the same or different aspects of depression. It has been proposed that using a combination of various depression scales could capture the primary and specific domains of depression more comprehensively [16]. Accordingly, we incorporated two scales into our analyses to better understand the dimensionality of both self-reported and clinician-assessed ratings.

Self-ratings are considered to be more susceptible to bias influenced by the severity of depression. For example, individuals with severe depression may downplay their symptoms, while those with milder depression might exaggerate them [17, 18].

Furthermore, certain aspects of psychopathology, such as retardation or hypochondriasis, are challenging to assess through self-reports alone, as they are primarily observable by an external evaluator [19]. Self-rating is particularly vulnerable to fixed response biases in some individuals, such as acquiescence bias, social desirability bias, or the exaggeration of symptoms in an effort to obtain better care [17, 19]. The accuracy of self-reporting relies on the individual’s educational background and capacity for introspection, and it can be influenced by the clinician’s expectations regarding the prescribed treatment, particularly in naturalistic, non-blinded settings [19, 20]. Despite these limitations, self-reports may still capture a distinct aspect of the individual’s experience [1]. Self-rating scales provide valuable additional insights for evaluating treatment and capturing the patient’s perspective on their illness and recovery, which cannot be assessed through observer ratings alone.

The three BDI-II components identified in this study—interpreted as “cognitive distortions”, “behavioral”, and “somatic complaints”—align with those observed in previous versions of the BDI by Shafer [16], who referred to them as a “negative attitude towards self” factor, a “performance impairment” factor, and a “somatic” factor. These components are also consistent with the results reported by Hautzinger [21], which described three factors: “performance impairment”, “negative self-perception”, and “physical symptoms”, as well as with the findings of Seemüller *et al*. [19], who identified factors labeled “negative perception of oneself”, “performance”, and “somatic”. It is important to note that Beck’s description of the “negative attitudes towards self” factor, which includes feelings of failure, guilt, punishment, self-dislike, and worthlessness, aligns with the first component identified in our analysis [22]. However, suicidality, which may represent the most extreme manifestation of negative self-perception, did not cluster with the other “negative self-perception” components.

Numerous studies have explored the component structure of the HAMD, particularly the original 17-item version, which has been the focus of extensive research. While some evidence suggests a relatively stable factor structure, not all studies support this finding [16, 23]. In our opinion, several methodological problems may have contributed to these discrepancies. First, inadequate sample size has frequently been a significant concern, with many studies involving fewer than 100 participants—an approach that poses a high risk of yielding unstable factor solutions [22, 24]. Additionally, differences in study populations, different methods for factor extraction, diverse criteria for factor rotation, and various rotational techniques could account for the inconsistencies between studies [25].

The four-factor solution proposed by Shafer’s meta-analysis was largely supported. It identifies a “depression factor” encompassing core depressive symptoms, along with an “anxiety factor”, a “sleep factor”, and a “somatic factor” [16]. In line with prior findings, the HAMD-21 demonstrated strong internal consistency (Cronbach’s alpha = 0.90).

Considering that the HAMD-21 and the BDI-II exhibit an approximately 50% overlap in symptoms, our study did not reveal a distinct separation into two separate component groups when both instruments were used simultaneously. In our combined analyses, nearly all items from the BDI-II, along with the core depressive symptoms from the HAMD-21, were aggregated into Component I. Component II, on the other hand, comprised four HAMD items: Insomnia (delayed), Agitation, Anxiety (somatic), and Insight. Furthermore, our study demonstrated significant positive correlations between paired symptoms from the BDI-II and HAMD-21. The strong alignment between self-ratings and clinician-rated scales is evident in the correlation of 0.88 between HAMD-21 and BDI-II, which contrasts with earlier published findings [19].

We are confident that our sample, in terms of demographic and disease characteristics, closely resembles the IBS patient population typically seen in outpatient clinics. The principal component analyses of the two frequently used instruments in this sample of IBS patients revealed three components for the BDI-II and four distinct components for the HAMD-21. However, due to high correlations between variables within the components, as well as a low percentage of non-redundant residuals and high communality, the factor validity appeared to be satisfactory for both the BDI-II and HAMD-21 scales.

Our study represents the pioneering effort to identify the dimensions of the BDI-II and the HAMD-21 in Georgian adult IBS population. However, there are limitations to consider when interpreting our results. Firstly, due to the relatively small sample size and the inclusion of participants from only one geographical area, our findings should be considered preliminary and may not be fully generalizable. Additionally, as our study focused exclusively on adults (18 years and older), further research is needed to explore these dimensions in younger populations. On the positive side, the strengths of our study include its prospective design, and simultaneous application of the two most commonly used depression scales, which ensures high-quality, reliable data and reduces susceptibility to recall bias.

The components identified in this study align with those found in previous research. Incorporating both the HAMD-21 and the BDI-II in a multimethod approach can offer a more thorough evaluation of depressive symptoms for several reasons.

Both scales measure depression but from slightly different angles, which can enhance the overall understanding of an individual’s depressive symptoms. The BDI-II, completed by the individual, provides valuable insights into their personal experience of depression. This self-report measure captures nuances in their emotional state and subjective experiences, focusing on cognitive and affective symptoms such as feelings of hopelessness and worthlessness, and is sensitive to mood changes. The HAMD-21 is a clinician-administered tool, benefiting from the clinician’s expertise in interpreting symptoms and providing a professional assessment of the severity of depression. It often assesses more severe symptoms and includes items related to physical symptoms of depression, such as sleep disturbances and appetite changes.

It is important to highlight the significance of cross-verification. Discrepancies between the scales might provide additional insights into the individual’s experience or highlight areas needing further exploration. This approach enables clinicians to develop comprehensive profiles of their patient’s conditions and evaluate the effectiveness of treatments as well as their reintegration into normal social activities.

## 5. Conclusion

The Beck Depression Inventory and the Hamilton Depression Rating Scale are complementary rather than redundant, highlighting the importance of using multiple measures for a comprehensive assessment of depression. Comparing results from both scales can help validate findings and ensure that depressive symptoms are consistently identified.

## Data Availability

The datasets generated during this study are available from the corresponding author upon reasonable request. All materials and reagents used in this research can be provided upon request. This study complies with ethical guidelines, and all necessary approvals for data sharing have been secured.

## Supplementary Material

Supplementary material associated with this article can be found, in the online version, at https://doi.org/10.31083/AP44020.
