# Supplementary material for: Factor Analysis of the BDI-II and HAMD-21 in Patients With Irritable Bowel Syndrome
Source: Alpha Psychiatry. 2025 Jun 19;26(3):44020. doi: 10.31083/AP44020 (PMC12231368; doi:10.31083/AP44020)
Supplement: Supplementary file 1 [file 2757-8038-26-3-44020-s1.docx]

**Supplemental Material 1.**

**Gastrointestinal Complaints Assessment Questionnaire**

**Patient Information**

Last Name, First Name: _____________________

Date: ___________________

**1. Dietary Habits**

- Do you follow a specific diet?

Yes / No

If yes, please describe:

- How regularly do you eat?

Irregular / Regular (consistent meal times)

2. **Food Preferences**

- Do you consume more refined foods (white bread, sweets, and processed foods) or foods rich in fiber (vegetables, whole grains)?

Mostly refined / Mostly high-fiber / Balanced

**3. Duration of Illness**

- How long have you considered yourself unwell due to gastrointestinal issues?

Less than 1 week / 1-4 weeks / 1-3 months / More than 3 months

**4. Abdominal Discomfort**

- Discomfort in the abdominal area:
- Duration: ________________________
- Symptoms: Discomfort, stomach pain, heaviness in the epigastrium after eating, nausea, heartburn, belching

Severe flatulence:

**5. Pain Patterns**

- Spasmodic abdominal pain:
- Duration: ________________________
- Frequency: ________________________
- Is there pain or discomfort in the abdomen associated with defecation?

Yes / No

- Does the pain go away after defecation?

Yes / No

Accompanying symptoms of abdominal pain and frequency:

- Bloating: ________________________
- Distension: ________________________
- Heaviness: ________________________
- Fullness: ________________________
- Hoarseness (every day - how many times a week?): ________________________
- Flatulence: ________________________
- Other: __________________________________

**6. Bowel Movement and Stool Patterns**

- Defecation disorders:
- Diarrhea: Duration _____________ and Frequency _____________
- Constipation: Duration _____________ and Frequency _____________
- Straining during defecation / feeling of incomplete emptying
- Alternating between diarrhea and constipation
- Change in stool passage:

Difficult / Rapid / Incomplete evacuation

- Do symptoms worsen after eating?

Yes / No

- Changes in stool form?

Yes / No (Please complete the Bristol Stool Form Scale: Type 1-7)

- Is there mucus in the stool?

Yes / No

**7. Additional Symptoms**

- Nausea or other symptoms (e.g., drowsiness, lower back pain):

**8. Use of Medications**

- Please indicate if you use any of the following medications:
- Proton pump inhibitors: Yes / No
- Histamine 2 blockers: Yes / No
- Prokinetics: Yes / No
- Probiotics: Yes / No
- Antispasmodics: Yes / No
- Laxatives: Yes / No
- Antidiarrheals: Yes / No
- Analgesics: Yes / No
- Antidepressants: Yes / No
